# Supplementary material for: Multi-resolution community detection in massive networks
Source: Sci Rep. 2016 Dec 13;6:38998. doi: 10.1038/srep38998 (PMC5154182; doi:10.1038/srep38998)
Supplement: Supplementary Material [file srep38998-s1.pdf]

Multi-resolution community detection in massive networks

Jihui Han[1,*], **Wei Li**[1,+], **and Weibing Deng**[1]

[1]Complexity Science Center and Institute of Particle Physics, Central China Normal University Wuhan, 430079, China

[*]jh@mails.ccnu.edu.cn

[+]liw@mail.ccnu.edu.cn

ABSTRACT

This is the supplementary material for the article “Multi-resolution community detection in massive networks”.

Illustration of the application of our algorithm to the karate club network

In order to explain the proposed algorithm, we provide a detailed description of each step by using the *Karate* network. The flowchart of our algorithm for the *Karate* network is shown in Fig. S1. The detection process consists of the following steps.

Step1: Similarity calculation. The *Karate* network is unweighted, and thus the structure similarity $s(u,v)$ between two adjacent nodes u and v can be calculated as[1]:

$$s(u,v) = \frac{|\Gamma(u) \cap \Gamma(v)|}{\sqrt{|\Gamma(u)| \cdot |\Gamma(v)|}}, \tag{1}$$

where $\Gamma(u)$ is the set containing u and its adjacent nodes. According to equation (1), the similarity of each pair of adjacent nodes is calculated. Then we get a weighted network, where the weights of edges are given by the similarities. As shown in Fig. S1 a→b.

Step2: Label propagation. Nodes are updated asynchronously in random order with the generalized update rule as follows:

$$L(u) = L\left(\arg\max_{C_k}\left\{\sum_{v\in C_k} w(u,v)\right\}\right) \tag{2}$$

where L denotes the label of a node or a subnetwork. C_k is the subnetwork containing a set of nodes connected to node u and with the same label k. $w(u,v)$ is the weight of edge (u,v). For self-loops, we rescale their weights to $\lambda \cdot w(u,v)$ (the resolution parameter λ is set to be 0.6 in this example). The above process is performed iteratively until there are no changes of labels. At the end of this step we should obtain some meta-communities (see Fig. S1 b→c, d→e, f→g).

Step3: Network aggregation. An aggregated (coarse-grained) network is built with its nodes representing the meta-communities detected in step 2. The weights of edges between the new nodes are given by the total number of edges between nodes in

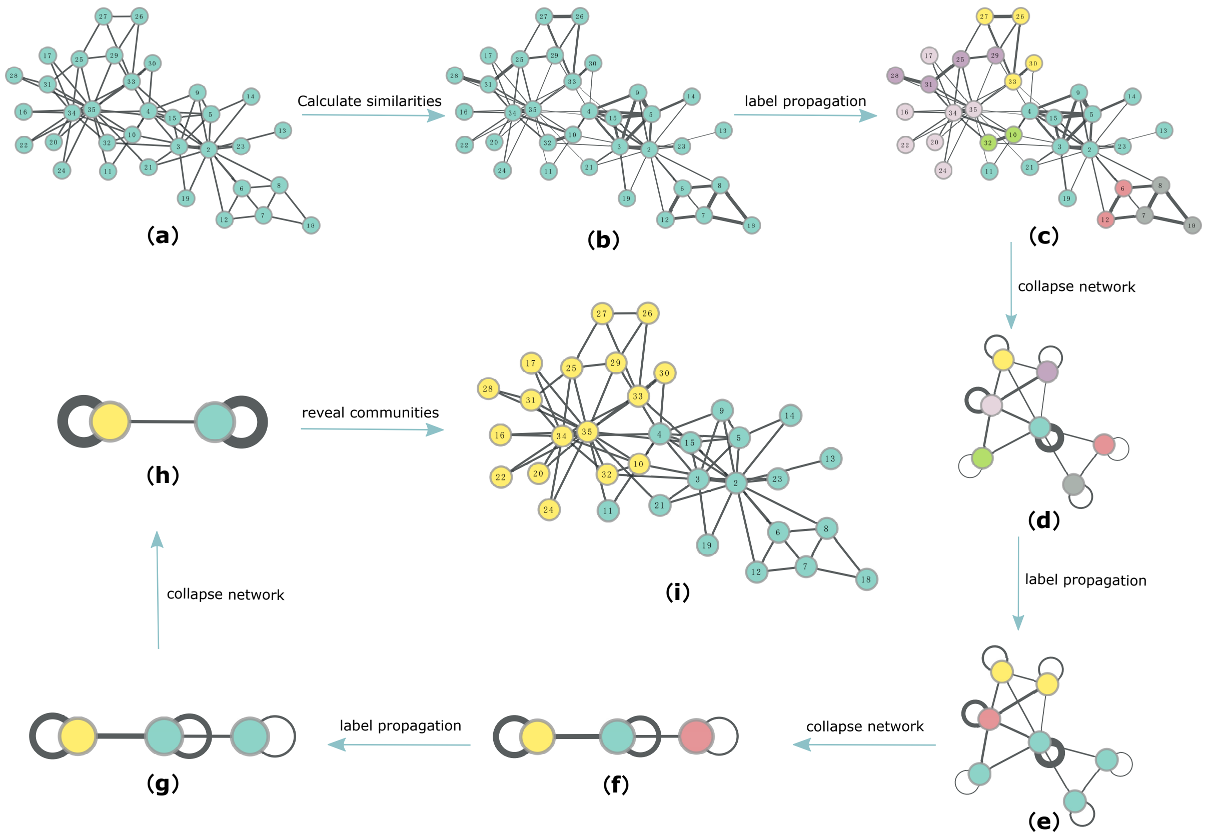

Supplementary Figure S1. (Colour online) **The flowchart of our algorithm for the karate club network.** The colours of nodes represent different meta-communities in all snapshots expect for (a) and (b). The line widths of edges indicate weights or similarities.

the corresponding two meta-communities. The number of edges between nodes of the same meta-community leads to the weight of self-loop for this meta-community in the new network (see Fig. S1 c→d, e→f, g→h).

Step4: Repeat steps 2 and 3 iteratively, until the number of meta-communities no longer changes. Then communities can be restored from the last stable aggregated network (see Fig. S1 h→i).

Additional analysis of our method

Figure S2 shows the number of meta-communities detected by our algorithm at each iteration on the real-world networks. As one can see, our algorithm generally converges after a small number of iterations, even on large networks. The number of meta-communities decreases dramatically in each iteration, and thus our algorithm converges fast.

Figure S3 shows the evolution of modularity during the detection process of our algorithm on the four real-world networks. In general, the modularity shows an increasing trend during the detection process, although we do not optimize modularity directly. This provides further experimental evidence for the effectiveness of our method. Note that the modularity does not always increase during our detection process. This implies that, in contrast to modularity optimization, in our method small

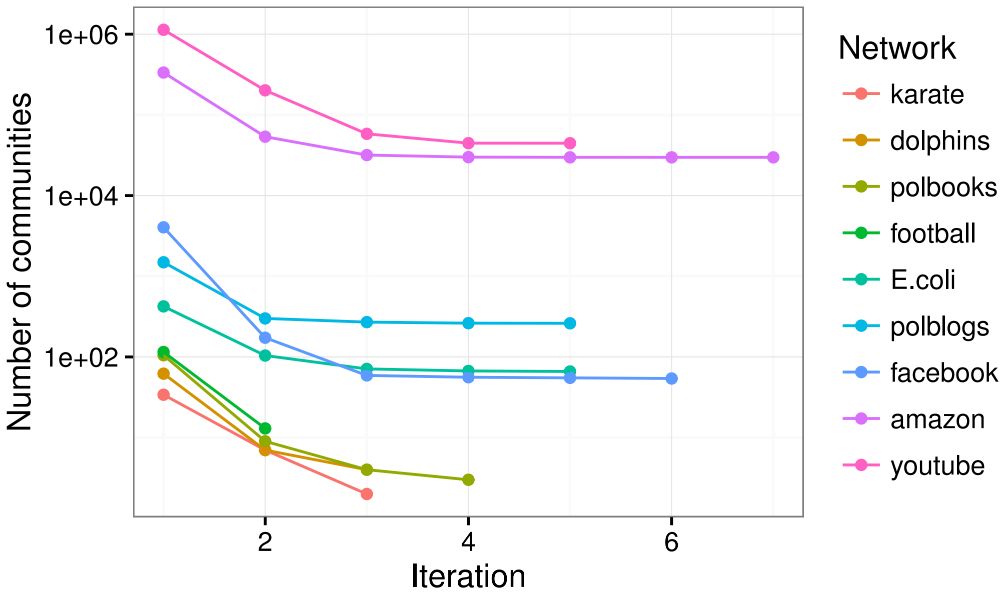

Supplementary Figure S2. (Colour online) The number of meta-communities detected by our method at each iteration on the real-world networks.

communities will not be merged so as to increase the modularity. This eliminates the resolution limit issue of modularity-based methods (see Fig. S7).

Additional benchmarks

Here, we included three more algorithms in our experiments. The full list of the algorithms is shown in Table S1. In Figures S4, S5 and S6, each point always corresponds to an average over 100 different network realizations.

Figure S4 shows the results of different algorithms on the GN benchmark[12]. Generally, modularity-based methods perform quite well, especially for SG and FADM. Our method obtains a reasonable performance and runs extremely fast.

Figure S5 shows the results of different algorithms on the LFR benchmark[13]. As can be seen, in contrast to the GN benchmark, modularity-based methods do not have remarkable performances on the LFR networks, and perform worse in the case of larger networks with smaller communities, due to the resolution limit of modularity[14]. Our algorithm performs fairly well on the LFR networks, and runs very fast, even faster than LP in some cases. Infomap performs the best in the case of larger networks. LE has rather poor performance and fails to find communities at low mixing parameters.

Figure S6 shows the number of communities detected by different algorithms as a function of average degree on ER and SF random networks. The GN algorithm is excluded because it is too slow to be used for analysis. In both cases, our method, LP and Infomap always find a single community containing all nodes of the network when connections are densely enough. However, the rest five methods: FG, FADM, Louvain, WT and LE, are not so good, as they always find a few communities even when the network is dense enough.

Figure S7 shows the results of different algorithms on networks that are made of some cliques connected by a single edge

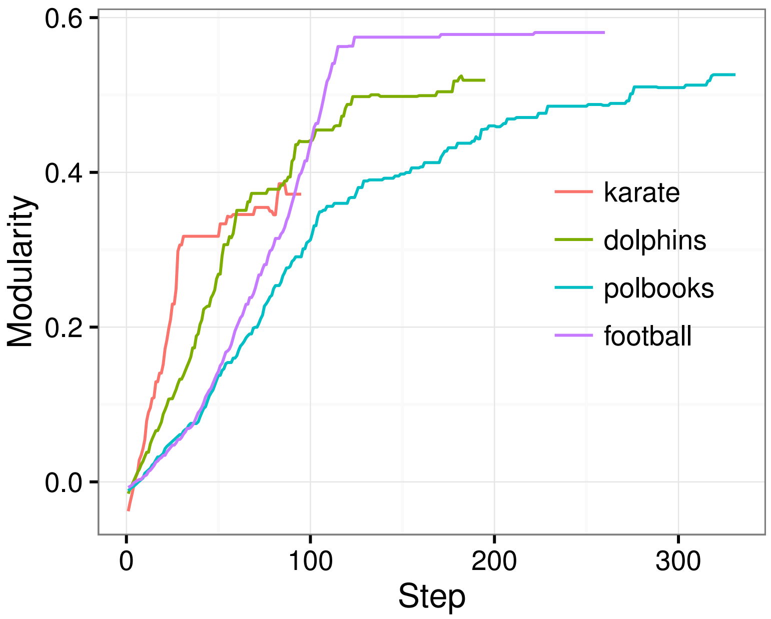

Supplementary Figure S3. (Colour online) The evolution of modularity during our detection process.

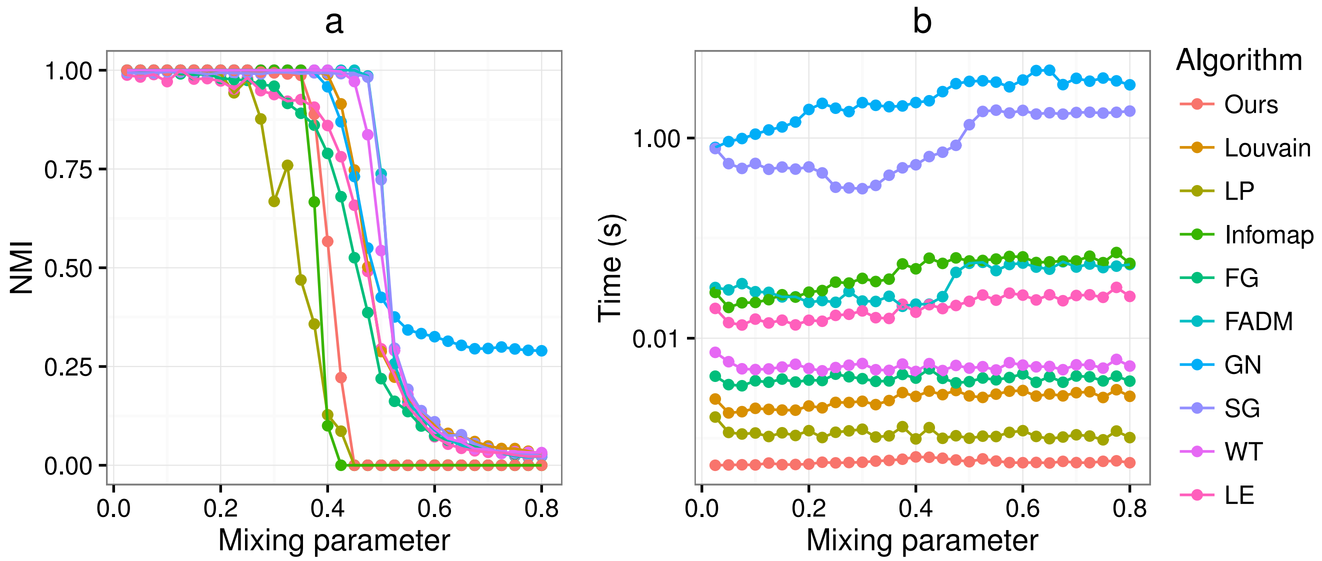

Supplementary Figure S4. (Colour online) **Tests of the algorithms on the GN benchmark.** (a) shows the normalized mutual information as a function of the mixing parameter. (b) shows the execution time of different algorithms on the benchmark.

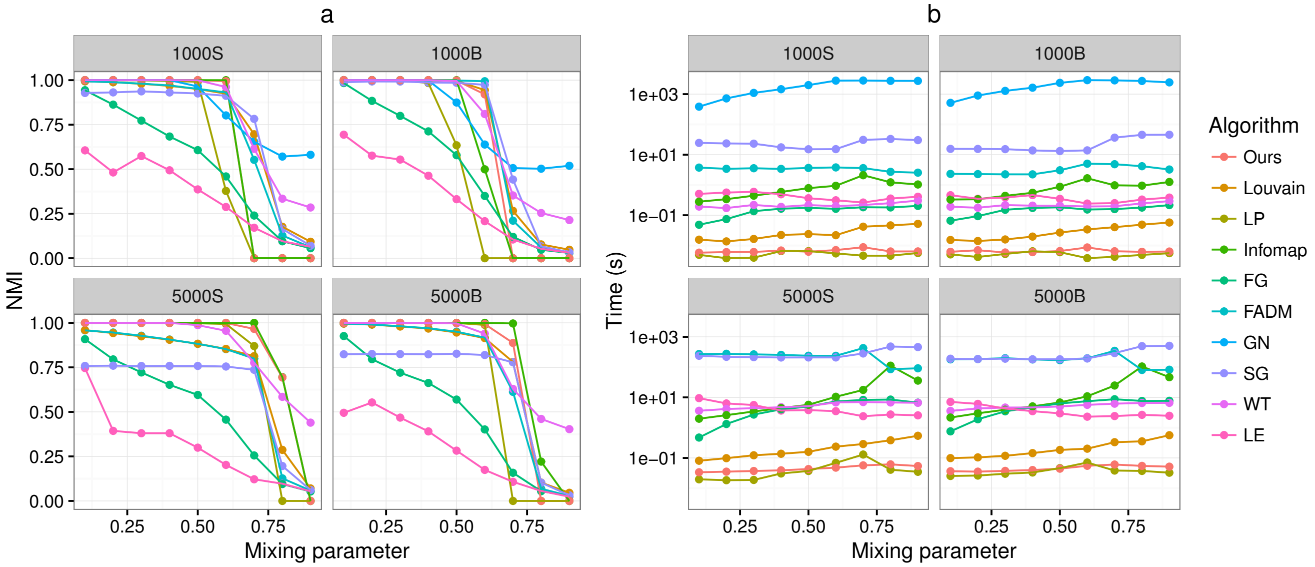

Supplementary Figure S5. (Colour online) **Tests of the algorithms on the LFR benchmark.** (a) shows the normalized mutual information as a function of the mixing parameter. (b) shows the execution time of different algorithms on the benchmark.

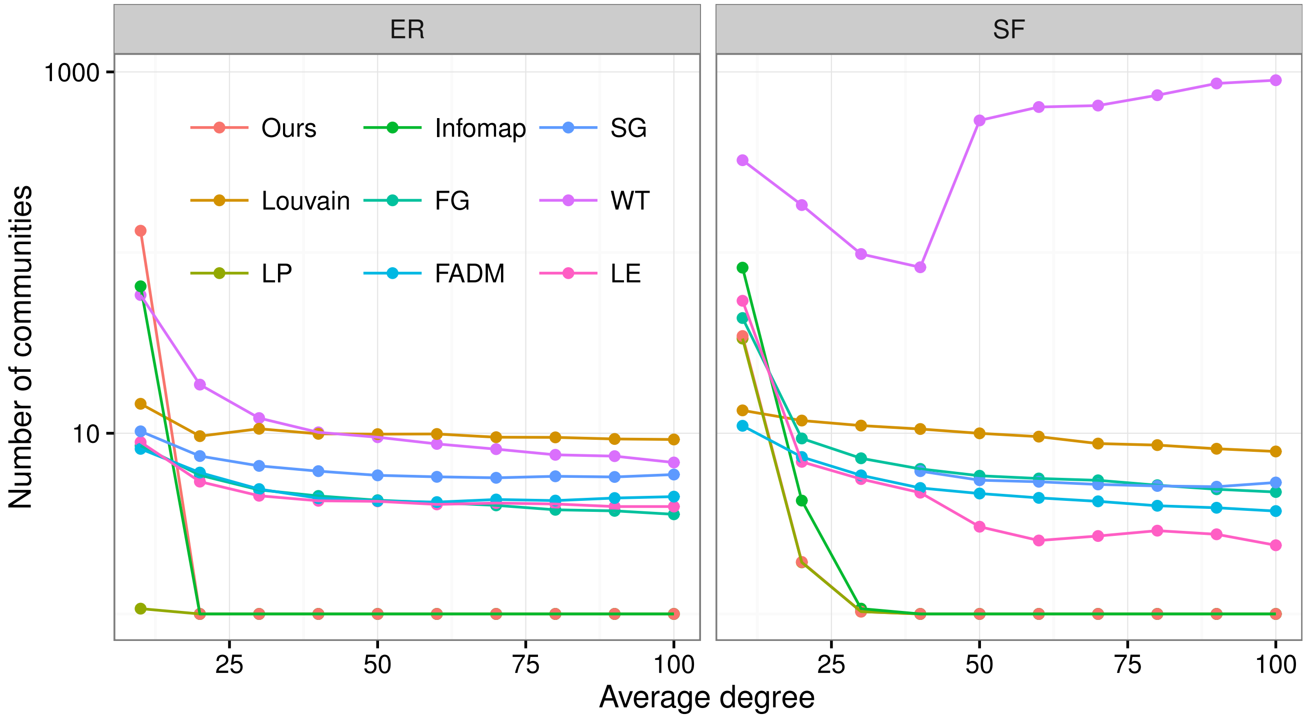

Supplementary Figure S6. (Colour online) **Tests of the algorithms on ER and SF random networks.** The plots show the number of communities detected by different algorithms as a function of the average degree.

Author and Ref.	Label	Time complexity
Blondel et al.[2]	Louvain	$O(m)$
Raghavan et al.[3,4]	LP (**L**abel **P**ropagation)	$O(m)$
Rosvall & Bergstrom[5]	Infomap	$O(m)$
Clauset et al.[4,6]	FG (**F**ast **G**reedy)	$O(n\log^2 n)$
Treviño III et al.[7]	FADM (**F**ast and **A**ccurate **D**etermination of **M**odularity)	$O(n^3)$
Girvan & Newman[4,8]	GN	$O(nm^2)$
Reichardt & Bornholdt[4,9]	SG (**S**pin **G**lass)	parameter dependent
Pascal & Matthieu[4,10]	WT (**W**alk **T**rap)	$O(n^2\log n)$
Newman[4,11]	LE (**L**eading **E**igenvector)	$O(n^2)$

Supplementary Table S1. List of the algorithms used in our additional benchmarks, where the first column indicates the designers and references, the second the label used to indicate the algorithm and the last the time complexity of the algorithm. In the last column, n and m denote the number of nodes and the number of edges in the network respectively.

with each other. As we can see, our method correctly finds all the predefined cliques in all cases.

Figure S8 shows the performances of different algorithms on the real-world networks. We can see that the performance of our method is competitive compared with other methods, and the time complexity of our method is fairly low, even lower than LP in most cases. The actual values of modularity, NMI and execution time are shown in Table S2.

Detailed results of the E. Coli network

We have analysed the E. Coli transcriptional network in detail. Figure S9 shows the modules identified by our algorithm with $\lambda = 1$, which include 5 isolated nodes, 26 modules with two operons, 9 modules with three operons and 22 modules with more than three operons. Table S3 lists the entire operon list of the 22 modules which have more than three operons.

Time complexity analysis

We have measured the speed of our algorithm by computing the time of analysing a LFR network of increasing size N, and compared the results with those of the other four fast methods: LP, FG, Louvain and Infomap. It was shown in Fig. S10 that, the computation time of all the five methods depends linearly on the network size. But our algorithm has a significantly lower time complexity than the rest of four methods, which makes it very efficient in dealing with large-scale networks.

Network	Metric	Ours	Louvain	LP	Infomap	FG	FADM	GN	SG	WT	LE
Karate	Time (s)	**0.0001**	0.0014	0.0010	0.0116	0.0029	0.0030	0.0070	0.7494	0.0020	0.0150
Karate	Modularity	0.3720	0.4188	0.3459	0.4020	0.3807	**0.4198**	0.4013	0.4194	0.3532	0.3934
Karate	NMI	**1.0000**	0.6884	0.6733	0.8207	0.8004	0.5878	0.5810	0.6093	0.4899	0.5792
Dolphins	Time (s)	**0.0001**	0.0014	0.0009	0.0205	0.0028	0.0091	0.0270	1.1589	0.0024	0.0238
Dolphins	Modularity	0.4080	0.5185	0.4785	0.5244	0.4955	**0.5277**	0.5194	0.5259	0.4888	0.4912
Dolphins	NMI	**0.8360**	0.4617	0.6517	0.5288	0.5571	0.5973	0.6256	0.5464	0.5816	0.5094
Polbooks	Time (s)	**0.0003**	0.0016	0.0009	0.0470	0.0031	0.0141	0.2454	2.6493	0.0036	0.0234
Polbooks	Modularity	0.4980	0.5205	0.4919	0.5228	0.5020	**0.5270**	0.5168	0.5257	0.5070	0.4672
Polbooks	NMI	**0.5760**	0.5121	0.5570	0.4935	0.5308	0.5705	0.5585	0.4967	0.5427	0.5201
Football	Time (s)	**0.0004**	0.0018	0.0009	0.0323	0.0037	0.0354	0.5323	0.9806	0.0041	0.0258
Football	Modularity	0.6030	**0.6046**	0.5895	0.6005	0.5497	**0.6046**	0.5996	0.6011	0.6029	0.4926
Football	NMI	0.9100	0.8903	0.8804	**0.9242**	0.6977	0.8903	0.8789	0.9051	0.8874	0.6987
Ecoli	Time (s)	**0.0007**	0.0026	0.0015	0.1492	0.0028	0.5702	0.5110		0.0082	0.1104
Ecoli	Modularity	0.7680	0.7953	0.7266	0.7586	**0.7962**	0.7698	0.7950		0.7542	0.7624
Polblogs	Time (s)	0.0190	0.0339	**0.0054**	1.2812	0.4264	0.6864	2377.8112		0.6169	0.1869
Polblogs	Modularity	0.4250	0.4271	0.4047	0.4225	**0.4270**	0.4259	0.4180		0.4256	0.4243
Polblogs	NMI	0.3791	0.3763	0.3727	0.3308	0.3782	**0.4805**	0.3035		0.3707	0.3786
Facebook	Time (s)	0.1528	0.1859	**0.0376**	9.9525	4.4235	133.7285	34739.0721	1385.0690	5.5856	2.9054
Facebook	Modularity	0.8130	0.8348	0.8135	0.8099	0.7774	**0.8354**	0.8149	0.8343	0.8119	0.7991
Amazon	Time (s)	**2.5690**	6.4800	68.8657	133.0000						
Amazon	Modularity	0.7710	**0.9260**	0.7548	0.7990						
Youtube	Time (s)	**14.4724**	38.5800	163.2239	488.0000						
Youtube	Modularity	0.6670	0.6848	0.5510	**0.6980**						

Supplementary Table S2. Detailed results of different algorithms on real-world networks. Each value is an average over 100 different runs. The best value of each row is highlighted in bold. Some of the values in the table are missing because the time complexity of the algorithm is too high, or the algorithm is not suitable for that particular network. In our algorithm, the resolution parameter is set to be 0.6.

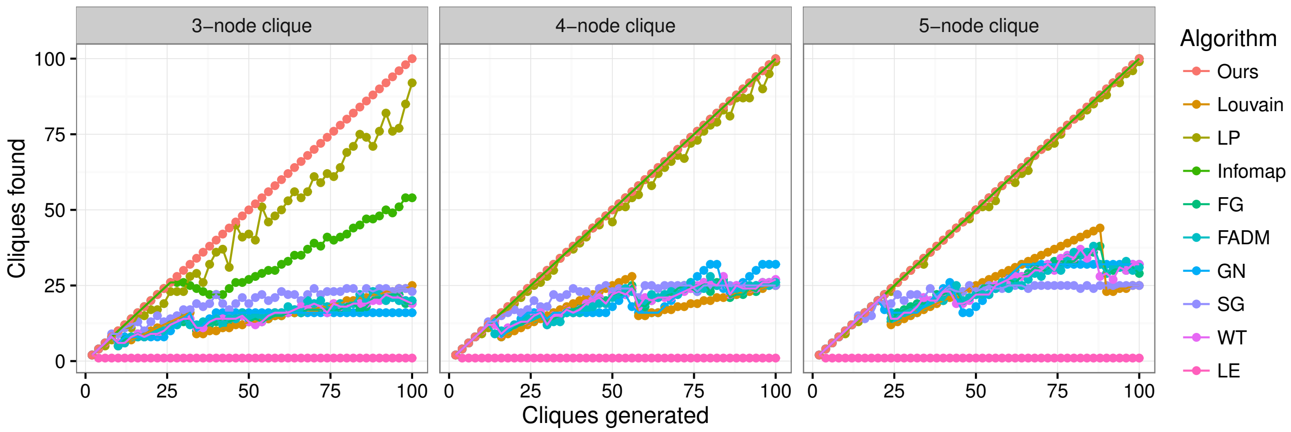

Supplementary Figure S7. (Colour online) Tests of the algorithms on networks comprised of identical cliques which are connected by single edges in a way that can be depicted as a ring.

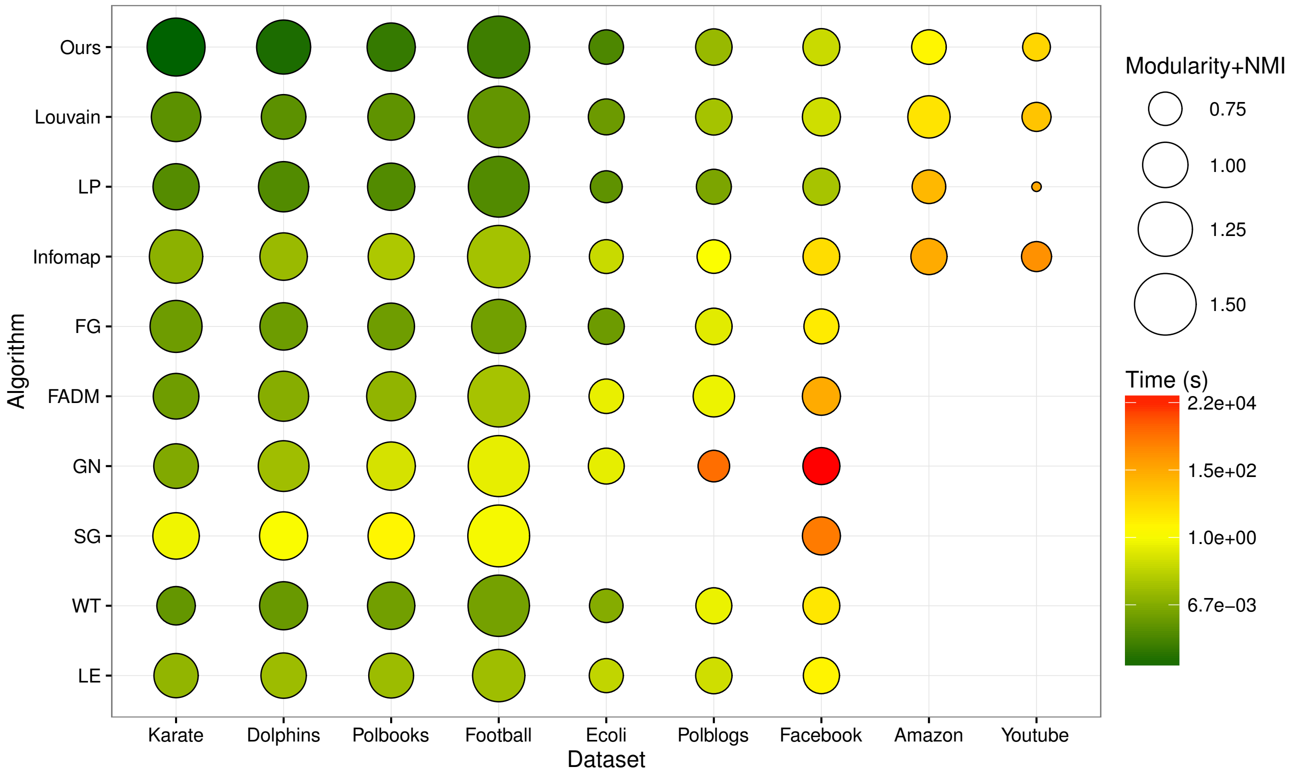

Supplementary Figure S8. (Colour online) **Performances of different algorithms on real-world networks.** The bubbles show the composite performance and execution time for different algorithms. The composite performance including two measures: NMI and modularity. For networks without ground truth partitions, we only show the modularity values. Each value is an average over 100 different runs. Some of the results are missing because the time complexity of the algorithm is too high, or the algorithm is not suitable for that particular network. In our algorithm, the resolution parameter is set to be 0.6.

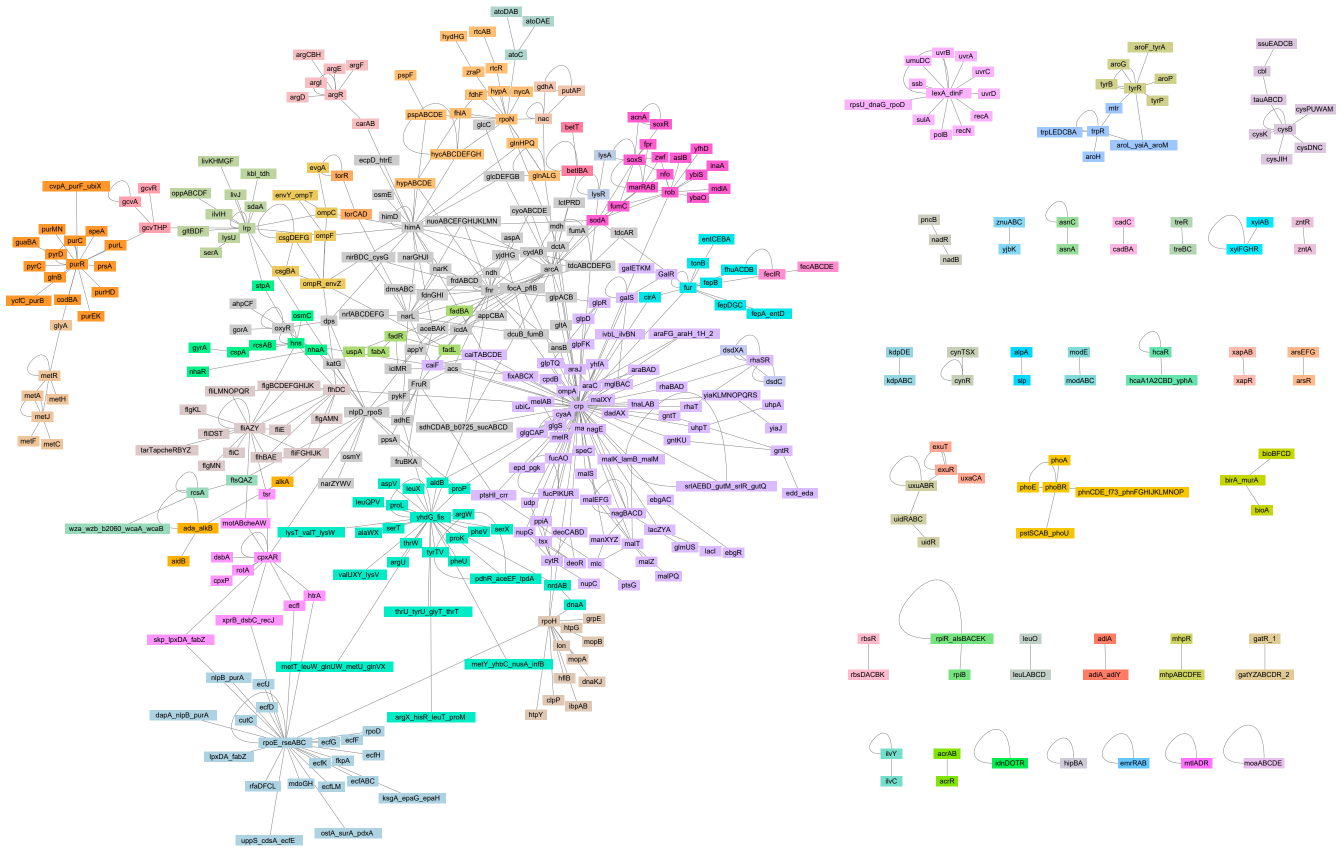

Supplementary Figure S9. (Colour online) **The modules detected by our algorithm on the E. Coli transcriptional regulatory network.** The colors of nodes indicate different detected modules.

Index	Operons
1	aceBAK, acs, adhE, ahpCF, ansB, appCBA, appY, arcA, aspA, cydAB, cyoABCDE, dctA, dcuB_fumB, dmsABC, dps, ecpD_htrE, fdnGHI, fnr, focA_pflB, frdABCD, fruBKA, FruR, fumA, glcC, glcDEFGB, glpACB, gltA, gorA, himA, himD, icdA, iclMR, katG, lctPRD, mdh, narGHJI, narK, narL, narZYWV, ndh, nirBDC_cysG, nlpD_rpoS, nrfABCDEFG, nuoABCEFGHIJKLMN, osmE, osmY, oxyR, ppsA, pykF, sdhCDAB_b0725_sucABCD, tdcABCDEFG, tdcAR, yjdHG
2	acnA, aslB, fpr, fumC, inaA, marRAB, mdlA, nfo, rob, sodA, soxR, soxS, ybaO, ybiS, yfhD, zwf
3	aroH, aroL_yaiA_aroM, mtr, trpLEDCBA, trpR
4	cbl, cysB, cysDNC, cysJIH, cysK, cysPUWAM, ssuEADCB, tauABCD
5	cirA, entCEBA, fepA_entD, fepB, fepDGC, fhuACDB, fur, tonB
6	acnA, aslB, fpr, fumC, inaA, marRAB, mdlA, nfo, rob, sodA, soxR, soxS, ybaO, ybiS, yfhD, zwf
7	clpP, dnaKJ, grpE, hflB, htpG, htpY, ibpAB, lon, mopA, mopB, rpoH
8	codBA, cvpA_purF_ubiX, glnB, guaBA, prsA, purC, purEK, purHD, purL, purMN, purR, pyrC, pyrD, speA, ycfC_purB
9	cpxAR, cpxP, dsbA, ecfI, htrA, motABcheAW, rotA, skp_lpxDA_fabZ, tsr, xprB_dsbC_recJ
10	csgBA, csgDEFG, envY_ompT, evgA, ompC, ompF, ompR_envZ
11	cspA, gyrA, hns, nhaA, nhaR, osmC, rcsAB, stpA
12	cutC, dapA_nlpB_purA, ecfABC, ecfD, ecfF, ecfG, ecfH, ecfJ, ecfK, ecfLM, fkpA, ksgA_epaG_epaH, lpxDA_fabZ, mdoGH, nlpB_purA, ostA_surA_pdxA, rfaDFCL, rpoD, rpoE_rseABC, uppS_cdsA_ecfE
13	fabA, fadBA, fadL, fadR, uspA
14	fdhF, fhlA, glnALG, glnHPQ, hycABCDEFGH, hydHG, hypA, hypABCDE, nycA, pspABCDE, pspF, rpoN, rtcAB, rtcR, zraP
15	flgAMN, flgBCDEFGHIJK, flgKL, flgMN, flhBAE, flhDC, fliAZY, fliC, fliDST, fliE, fliFGHIJK, fliLMNOPQR, tarTapcheRBYZ
16	gltBDF, ilvIH, kbl_tdh, livJ, livKHMGF, lrp, lysU, oppABCDF, sdaA, serA
17	glyA, metA, metC, metF, metH, metJ, metR
18	lexA_dinF, polB, recA, recN, rpsU_dnaG_rpoD, ssb, sulA, umuDC, uvrA, uvrB, uvrC, uvrD
19	phnCDE_f73_phnFGHIJKLMNOP, phoA, phoBR, phoE, pstSCAB_phoU
20	alaWX, aldB, argU, argW, argX_hisR_leuT_proM, aspV, dnaA, leuQPV, leuX, lysT_valT_lysW, metT_leuW_glnUW_metU_glnVX, metY_yhbC_nusA_infB, nrdAB, pdhR_aceEF_lpdA, pheU, pheV, proK, proL, proP, serT, serX, thrU_tyrU_glyT_thrT, thrW, tyrTV, valUXY_lysV, yhdG_fis
21	araBAD, araC, araE, araFG_araH_1H_2, araJ, caiF, caiTABCDE, cpdB, crp, cyaA, cytR, dadAX, deoCABD, deoR, ebgAC, ebgR, edd_eda, epd_pgk, fixABCX, fucAO, fucPIKUR, galETKM, GalR, galS, glgCAP, glgS, glmUS, glpD, glpFK, glpR, glpTQ, gntKU, gntR, gntT, ivbL_ilvBN, lacI, lacZYA, malEFG, malI, malK_lamB_malM, malPQ, malS, malT, malXY, malZ, manXYZ, melAB, melR, mglBAC, mlc, nagBACD, nagE, nupC, nupG, ompA, ppiA, ptsG, ptsHI_crr, rhaBAD, rhaSR, rhaT, speC, srlAEBD_gutM_srlR_gutQ, tnaLAB, tsx, ubiG, udp, uhpA, uhpT, yhfA, yiaJ, yiaKLMNOPQRS
22	argCBH, argD, argE, argF, argI, argR, carAB

Supplementary Table S3. The operon list of the 22 detected modules which have more than 3 operons in the E. Coli network.

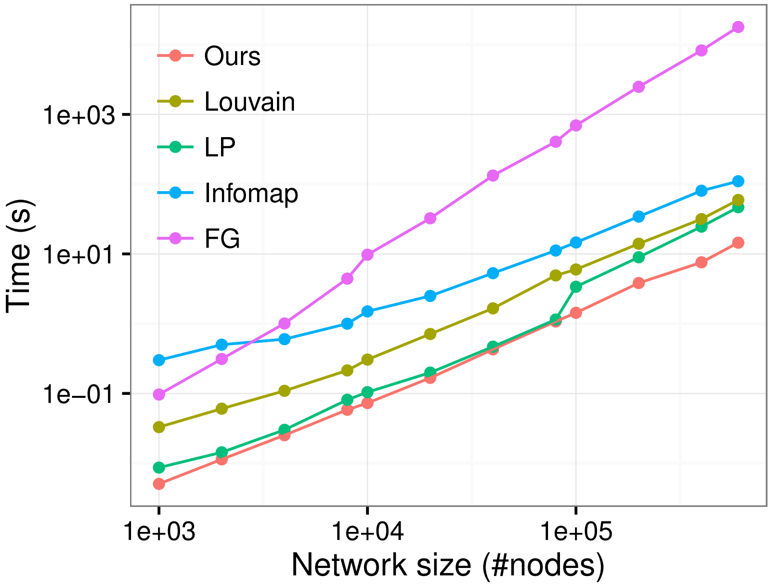

Supplementary Figure S10. (Colour online) **The average time (in seconds) consumed to analyse a LFR network of increasing size** N**.** The parameters of the networks are: average degree $\langle k \rangle = 20$, maximum degree $k_{max} = 100$, exponents of the power-laws are $\tau_1 = 2$ for degree distribution and $\tau_2 = 1$ for community size distribution, and communities have nodes from 20 to 200. Each data point corresponds to an average over 30 different runs.

References

1. Huang, J., Sun, H., Han, J. & Feng, B. Density-based shrinkage for revealing hierarchical and overlapping community structure in networks. *Physica A: Statistical Mechanics and its Applications* **390**, 2160 – 2171 (2011).

2. Blondel, V. D., Guillaume, J.-L., Lambiotte, R. & Lefebvre, E. Fast unfolding of communities in large networks. *Journal of Statistical Mechanics: Theory and Experiment* **2008**, P10008 (2008).

3. Raghavan, U. N., Albert, R. & Kumara, S. Near linear time algorithm to detect community structures in large-scale networks. *Phys. Rev. E* **76**, 036106 (2007).

4. Csardi, G. & Nepusz, T. The igraph software package for complex network research. *InterJournal* **Complex Systems**, 1695 (2006). URL http://igraph.org.

5. Rosvall, M. & Bergstrom, C. T. Maps of random walks on complex networks reveal community structure. *Proceedings of the National Academy of Sciences* **105**, 1118–1123 (2008).

6. Clauset, A., Newman, M. E. J. & Moore, C. Finding community structure in very large networks. *Phys. Rev. E* **70**, 066111 (2004).

7. III, S. T., Nyberg, A., Genio, C. I. D. & Bassler, K. E. Fast and accurate determination of modularity and its effect size. *Journal of Statistical Mechanics: Theory and Experiment* **2015**, P02003 (2015).

8. Newman, M. E. J. & Girvan, M. Finding and evaluating community structure in networks. *Phys. Rev. E* **69**, 026113 (2004).

9. Reichardt, J. & Bornholdt, S. Detecting fuzzy community structures in complex networks with a potts model. *Phys. Rev. Lett.* **93**, 218701 (2004). URL http://link.aps.org/doi/10.1103/PhysRevLett.93.218701.

10. Pons, P. & Latapy, M. *Computing Communities in Large Networks Using Random Walks*, 284–293 (Springer Berlin Heidelberg, Berlin, Heidelberg, 2005). URL http://dx.doi.org/10.1007/11569596_31.

11. Newman, M. E. J. Finding community structure in networks using the eigenvectors of matrices. *Phys. Rev. E* **74**, 036104 (2006).

12. Girvan, M. & Newman, M. E. J. Community structure in social and biological networks. *Proceedings of the National Academy of Sciences* **99**, 7821–7826 (2002).

13. Lancichinetti, A., Fortunato, S. & Radicchi, F. Benchmark graphs for testing community detection algorithms. *Phys. Rev. E* **78**, 046110 (2008).

14. Fortunato, S. & Barthélemy, M. Resolution limit in community detection. *Proceedings of the National Academy of Sciences* **104**, 36–41 (2007).
